# Evaluation of methods for volumetric analysis of pediatric brain data: The child**metrix** pipeline versus adult-based approaches

**DOI:** 10.1016/j.nicl.2018.05.030

**Published:** 2018-05-23

**Authors:** Thanh Vân Phan, Diana M. Sima, Caroline Beelen, Jolijn Vanderauwera, Dirk Smeets, Maaike Vandermosten

**Affiliations:** a ico**metrix**, Research and Development, Leuven, Belgium; b Experimental Oto-rhino-laryngology, Department Neurosciences, KU Leuven, Leuven, Belgium; c Parenting and Special Education Research Unit, Faculty of Psychology and Educational Science, KU Leuven, Leuven, Belgium

**Keywords:** Brain volumetric analysis, Neuroimaging data, Pediatric atlas, Child-adjusted processing, Magnetic resonance imaging

## Abstract

Pediatric brain volumetric analysis based on Magnetic Resonance Imaging (MRI) is of particular interest in order to understand the typical brain development and to characterize neurodevelopmental disorders at an early age. However, it has been shown that the results can be biased due to head motion, inherent to pediatric data, and due to the use of methods based on adult brain data that are not able to accurately model the anatomical disparity of pediatric brains. To overcome these issues, we proposed child**metrix**, a tool developed for the analysis of pediatric neuroimaging data that uses an age-specific atlas and a probabilistic model-based approach in order to segment the gray matter (GM) and white matter (WM). The tool was extensively validated on 55 scans of children between 5 and 6 years old (including 13 children with developmental dyslexia) and 10 pairs of test-retest scans of children between 6 and 8 years old and compared with two state-of-the-art methods using an adult atlas, namely ico**brain** (applying a probabilistic model-based segmentation) and Freesurfer (applying a surface model-based segmentation). The results obtained with child**metrix** showed a better reproducibility of GM and WM segmentations and a better robustness to head motion in the estimation of GM volume compared to Freesurfer. Evaluated on two subjects, child**metrix** showed good accuracy with 82–84% overlap with manual segmentation for both GM and WM, thereby outperforming the adult-based methods (icobrain and Freesurfer), especially for the subject with poor quality data. We also demonstrated that the adult-based methods needed double the number of subjects to detect significant morphological differences between dyslexics and typical readers. Once further developed and validated, we believe that child**metrix** would provide appropriate and reliable measures for the examination of children's brain.

## Highlights

• Brain segmentation is often biased when analyzing children's brain MRI.
• child**metrix** is an automatic tool developed for segmentation of pediatric brain MRI.
• child**metrix** shows good accuracy, reproducibility and robustness to head motion.
• child**metrix** outperforms adult-based methods when applied on pediatric data.
• A better segmentation is reflected in a higher sensitivity for clinical differences.

## 1 Introduction

Brain volumetric analyses have been performed in many Magnetic Resonance Imaging (MRI)-based studies on typical brain development and neurodevelopmental disorders (Anderson et al., 2012; Holland et al., 2014; Hu et al., 2013; Krogsrud et al., 2014; Nie et al., 2013). Investigating how volumetric measurements of the pediatric brains relate to behavioral measures and how they differ between groups can aid in understanding the neural etiology of a disorder. In order to quantify the structural anatomy of the brain, volume measurements are extracted by segmenting anatomical MRI scans, which are typically T1-weighted images. Although manual segmentation is still considered as the gold standard, this procedure is subject to inter- and intra-rater variability and its application is rather limited for population-based studies or clinical practice, since it requires time investment and excellent anatomical expertise. Hence, automated methods have been developed to address issues introduced by the processing of large amounts of data. Examples of the most commonly used software tools for automated brain segmentation are the FSL software packages (Jenkinson et al., 2012; Smith, 2002; Zhang et al., 2001), Statistical Parameters Mapping (SPM; Ashburner and Friston, 2000) and Freesurfer (Fischl et al., 2002). However, in the analysis of pediatric data, there are two main issues that automated methods should be able to overcome (Phan et al., 2017).

The first main issue is head motion that is typically present in pediatric data (Levman and Takahashi, 2015; Theys et al., 2014). Head motion generally results in blurring and ringing artifacts in MRI scans, which hinders the identification of tissue boundaries. Even subtle motion that is not easily detected by visual inspection has been shown to lead to systematic biases in automatic measurement of structural brain properties (Alexander-Bloch et al., 2016; Blumenthal et al., 2002; Reuter et al., 2015; Yendiki et al., 2014), leading to errors that are comparable to yearly atrophy rates in neurodegenerative diseases (Anderson et al., 2012; Barkhof et al., 2009; Rosas et al., 2011) or comparable to yearly growth rates of normal developing brain tissues (Hedman et al., 2012). Therefore, motion artifacts should be taken into account in order to control for the bias in the results.

The second main issue is that segmentation methods of popular software tools are based on a brain template (i.e. average intensity image or surface model) that is generally created from adult brain data. However, these adult brain templates might not be appropriate to model pediatric brains due to a non-linear and region-specific brain development, leading to a significant disparity between the pediatric and adult brains (Muzik et al., 2000; Yoon et al., 2009). For instance, Muzik et al. (2000) demonstrated results that preclude the application of SPM in children <6 years old, due to the error associated with spatial normalization of pediatric brain to an adult template. Mayer et al. (2016) concluded that limited agreement with ground truth was achieved when using FAST (software packages of FSL) and SPM for measuring the intracranial volumes and total brain volumes in children between 2 and 3 years of age. Hence, these findings suggest that the segmentation approach has to be adjusted to pediatric data, notably with the use of age-specific atlases instead of standard adult template.

In infants, efforts have been made for building age-specific atlases in order to deal with the significant anatomical disparity and the inverted MRI-contrast in infant brains relative to adult brains (Altaye et al., 2008; Fillmore et al., 2015; Fonov et al., 2009; Gousias et al., 2008; Kuklisova-Murgasova et al., 2011; Makropoulos et al., 2016; Oishi et al., 2008; Sanchez et al., 2012; Shi et al., 2011). In addition, tools have been developed in order to adapt brain segmentation to infant populations, such as ALFA (Serag et al., 2016), iBEAT (Dai et al., 2013) and AdAPT (Cardoso et al., 2013). Such initiatives were less applied for children from 4 years old onwards, given the assumption that standard software tools work well on pediatric populations from that age. However, weak consistency in the brain segmentation of older children (6–11 years old) was reported when using standard software tools such as FSL and Freesurfer (Schoemaker et al., 2016). Although pediatric atlases have been built for more accurate segmentation (Fonov et al., 2009), the potential improvement introduced by these atlases was not extensively validated. Therefore, it remains to be investigated and validated whether improved segmentation is observed when using these pediatric atlases and which property drives the improvement, such as the age range or the study-specificity of the atlas.

In this study, we propose a tool adapted to the pediatric population, called child**metrix**, which applies a probabilistic model-based segmentation using an age-matched pediatric atlas in order to segment the whole brain into gray matter (GM) and white matter (WM), and to estimate the tissue volume. This study further aims to extensively validate the proposed tool for brain segmentation of children in prepuberty (5–8 years old). At this age, the brain shows similar contrast with the adult brain on T1-weighted images (which is not the case in infants) but the anatomy is still substantially different due to non-linear and region-specific developmental trajectory of brain structures (Brain Development Cooperative Group, 2012; Hedman et al., 2012; Mills et al., 2016). Moreover, it is particularly interesting for studies investigating learning process during typical and atypical development because this age corresponds to a time in development when children start to acquire knowledge at school. For the validation, child**metrix** is compared to two state-of-the-art methods, ico**brain** (using probabilistic model-based segmentation) and Freesurfer (using surface model-based segmentation), that are both based on an adult atlas. Evaluation of each segmentation tool is based on the reproducibility, the segmentation accuracy and the robustness to motion and low image quality. Finally, we investigated the impact of using a child-adjusted method on further statistical analyses, such as group comparison.

## 2 Materials and methods

### 2.1 Pediatric MRI data

#### 2.1.1 Dataset description

The first pediatric dataset used in order to evaluate the proposed method is part of the Dyslexia Research Collaboration (DYSCO) project (Vanderauwera et al., 2017, Vanderauwera et al., 2016; Vandermosten et al., 2017, Vandermosten et al., 2015). Children were prepared before the scanning session with child-friendly protocols (Theys et al., 2014). T1-weighted images were acquired on a 3 T scanner (Philips, Best, The Netherlands) with 32-channel head coil using 3D Turbo field echo acquisition. The scanning parameters were as following: TR = 9.6 ms, TE = 3.6 ms, flip angle = 8°, FOV = 250 × 250 × 218 mm^3^, voxel size = 1 × 1 × 1.2 mm^3^, acquisition time = 6:22 min. As part of a longitudinal data collection, we used 72 T1-weighted images acquired when children were in kindergarten (73.9 ± 3.3 months old), of which 39 children had a family risk for dyslexia, defined by having a first degree relative with dyslexia. Based on longitudinal reading and spelling data acquired in second and third grade, 18 children were retrospectively classified as dyslexic (for more details on the diagnostic criteria see Vanderauwera et al., 2016). This study was approved by the ethics committee at the University Hospital of Leuven. The parents of the participants gave their written consent for the participation of the children in this study, in line with the Declaration of Helsinki.

In order to evaluate the reproducibility, we also used the Nathan-Kline Institute (NKI)-Rockland test-retest pediatric samples (Zuo et al., 2014). Test and retest scans are two scans acquired in a short period of time for which no significant changes are expected (typically at the same scanning session). The NKI dataset contains multimodal MRI scans, including T1-weighted images acquired on 3T scanner (SIEMENS MAGNETOM Trio Tim) with MPRAGE sequence. The scanning parameters used were: TR = 1900 ms, TE = 2.52 ms, flip angle = 9°, FOV = 256 × 256 × 176 mm^3^, voxel size = 1 × 1 × 1 mm^3^. Thirteen subjects aged between 6 and 8 years old (for which test-retest scans of T1-weighted images were available) were selected in order to represent the population that is the most similar to the children in kindergarten retrieved from the DYSCO project.

#### 2.1.2 Quality assessment

**Fig. 1:**
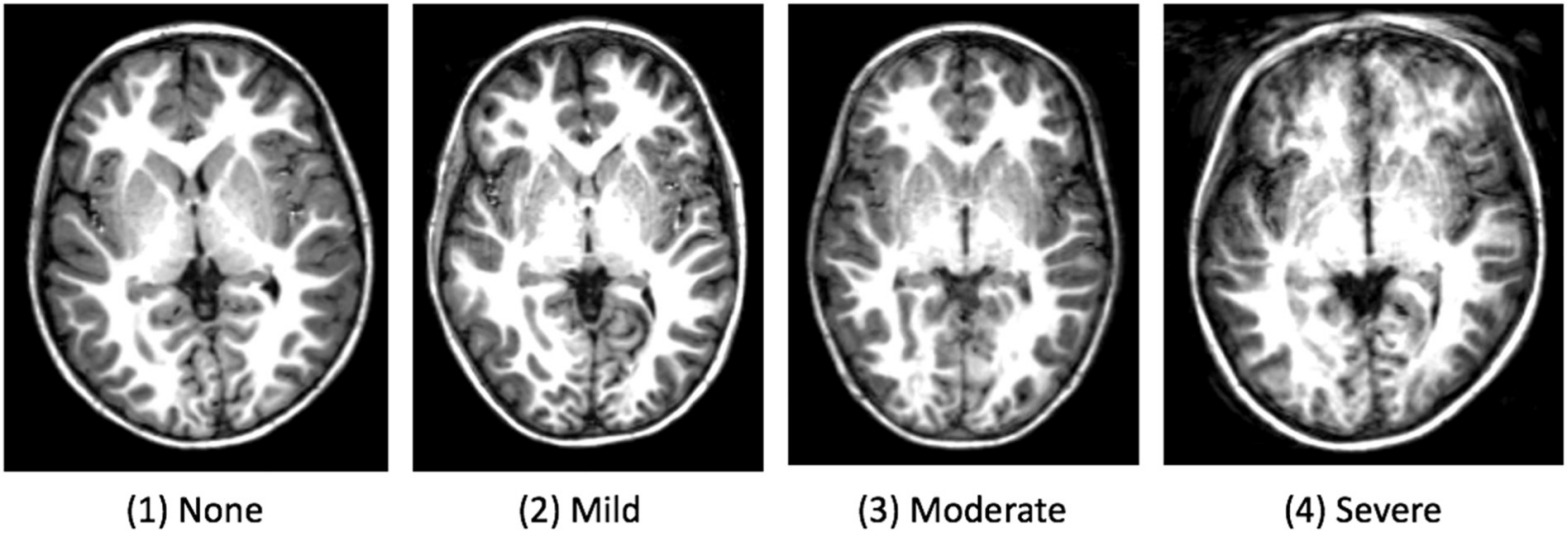
Examples of scans in each category according to Blumenthal's motion rating (Blumenthal et al., 2002): (1) “none” corresponding to little or no visible motion artifacts, (2) “mild” to enough detectable motion shown as subtle ringing, (3) “moderate” to significant ringing and (4) “severe” to extreme motion that renders the scan unusable.

All T1-weighted images were assessed for image quality based on the noise and motion artifacts. To assess head motion in the image, scans were visually graded in four categories according to Blumenthal's motion rating (Blumenthal et al., 2002), such as in illustrated in Fig. 1.

In addition, a quantitative assessment was performed to assess the noise by computing the signal-to-noise (SNR) and contrast-to-noise (CNR).

• The **SNR** is defined as the ratio of the mean of the signal intensity distribution measured in the white matter to the standard deviation of the noise intensity distribution multiplied by a factor 0.8, which is applied to compensate for Rayleigh distribution effect in the background noise (Gedamu et al., 2008). A good image quality corresponds to a high SNR.
• The **CNR** is based on the difference between the average of the intensity of distribution in the white matter and gray matter values divided by the standard deviation of the noise intensity distribution (Magnotta et al., 2006). A good image quality corresponds to a high CNR.

### 2.2 Childmetrix: segmentation method adjusted to children

The child**metrix** pipeline aims at computing brain structure volumes from pediatric data. It consists of several sub-pipelines performing image processing tasks. The pipeline is built in order to segment and to extract the whole brain volumes of GM and WM from 3D T1-weighted images of children. In order to adjust the brain segmentation to children, the volumes are computed with a probabilistic brain model optimized with expectation-maximization (EM) algorithm (Van Leemput et al., 1999) based on an age-specific atlas.

#### 2.2.1 Age-specific atlas

In the child**metrix** pipeline, the age-specific atlas is by default an independent pediatric population-based atlas, freely available on the website of Montreal Neurological Institute (MNI; http://www.bic.mni.mcgill.ca/). The atlas generally consists of the brain template (average grayscale image), the brain mask and tissue prior probability maps that are necessary for the EM segmentation. In order to segment brains of children from the DYSCO and NKI datasets, the atlas of children in prepuberty from the National Institutes of Health (NIH) pediatric database was selected and was here referred to as **NIHPD 4-8 atlas**. The atlas was built by non-linearly averaging brain images from 82 healthy children between 4.5 and 8.5 years old, recruited in the NIH-funded MRI study of normal brain development (Fonov et al., 2011). The brain template was iteratively updated by each brain image until convergence, in order to obtain a template for which the transformations (to map the template to each subject) and the intensity difference (between the template and each subject) were minimized. For this study, the asymmetric template is used since the tissue volumes are measured at the whole brain level (and not in each hemisphere). Note that it is possible to use another age-specific atlas such as another available pediatric atlas from MNI or a house-built study-specific atlas such as proposed in the Supplementary material.

#### 2.2.2 Brain segmentation pipeline

**Fig. 2:**
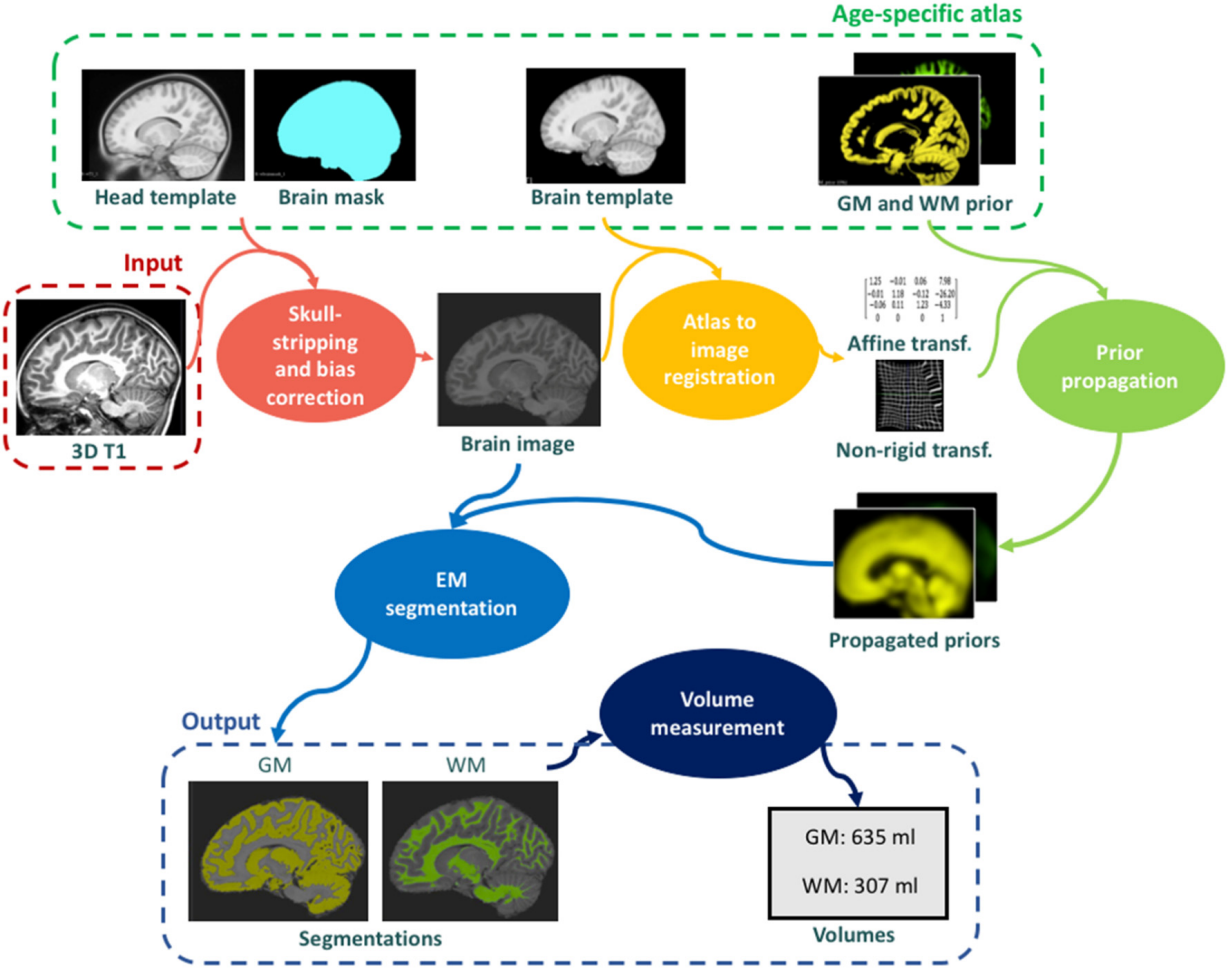
Scheme of brain volumetry pipeline in child**metrix**. In the pipeline, an age-specific atlas (e.g. NIHPD 4-8 atlas) is used in order to segment the pediatric brain MRI into gray matter (GM) and white matter (WM) with the expectation-maximization (EM) segmentation algorithm.

The brain segmentation pipeline of child**metrix** enables to extract the volumes of the two main brain tissues: gray matter (GM) and white matter (WM). For this application, the atlas used in the pipeline contained a head and a brain template, the corresponding brain mask, and prior probability maps of GM and WM. In order to adjust the segmentation method to children, the age-specific atlas described in Section 2.3.1 is used. The segmentation of the brain is performed with the 5 following steps (see Fig. 2). In the first step, the image is skull-stripped (i.e. removing non-brain tissues) and bias corrected (i.e. removing intensity non-uniformities). To do so, the head template of the atlas is registered to the target image to extract the affine and non-rigid transformations using NiftyReg (Modat et al., 2010; Ourselin et al., 2000). The brain mask of the atlas is then warped to the target image space (also called native space) by applying the transformations previously computed. The intensity non-uniformities are corrected based on the intensity distribution in the region defined by the brain mask, using the N4 bias field correction of ANTS (Tustison et al., 2010). In the second step, the brain template of the atlas is registered to the brain image in order to extract the affine and non-rigid transformations, using NiftyReg. In the third step, the tissue probability maps defined in the atlas space are then propagated to the native space by applying the transformations computed in the previous step. In the fourth step, the EM segmentation is performed on the target image using NiftySeg (Cardoso, 2012; Cardoso et al., 2013). The segmentations are obtained by the formulation and optimization of a Gaussian Mixture Model that takes into account the image intensities, the spatial prior knowledge of the tissues, the intensity non-uniformities caused by the bias field, and the spatial consistency based on Markov Random Field (MRF). The tissue probability maps of GM and WM act as prior knowledge in the adaptive relaxation EM algorithm (Cardoso et al., 2011). The tissue classes parameters and bias field parameters are iteratively estimated with the EM algorithm until convergence, with the spatial consistency being maintained. Segmentations of brain tissues are then obtained as probability maps that represent the fraction of tissue type at each voxel. In the fifth step, the tissue volumes are estimated by summing the tissue probability of each voxel and then multiplying the sum by the voxel volume.

### 2.3 Brain segmentation methods using an adult atlas

In order to validate child**metri**x for GM and WM segmentation in children (here, between 5 and 8 years old), we compared the method performance with two brain segmentation methods that use an adult atlas; ico**brain** and Freesurfer. ico**brain** uses the same probabilistic model-based segmentation as child**metrix** (Jain et al., 2015; Smeets et al., 2016) whereas Freesurfer uses a surface model-based segmentation (Dale et al., 1999; Fischl et al., 2002).

#### 2.3.1 icobrain: probabilistic model-based segmentation using an adult atlas

The cross-sectional pipeline of ico**brain** version 2.1.1, also known as MS**metrix**, computes the segmentation of the three main brain tissues (i.e. GM, WM and CSF) from T1-weighted images, in particular for patients with multiple sclerosis (Jain et al., 2015; Smeets et al., 2016). The segmentation method is based on a Gaussian Mixture Model, optimized with an expectation maximization algorithm (Van Leemput et al., 1999) for which the implementation is provided by NiftySeg (Cardoso, 2012; Cardoso et al., 2013). The different steps of the brain segmentation pipeline were described in more details in Section 2.3.2, since the segmentation method is the same as used in child**metrix** but with adult atlases as reference instead of the age-specific atlas. For the brain extraction, the head template used is the ICBM152 atlas and for the brain segmentation, the brain template is the Collin27 atlas, both freely available from MNI (http://www.bic.mni.mcgill.ca/). The ICBM152 atlas is built from 152 structural images of young adults (Fonov et al., 2009) and the Collin27 atlas corresponds to an average of 27 high quality T1 scans of the same normal adult subject, acquired on a 1.5T MRI scanner (Holmes et al., 1998). Tissue priors for CSF, GM and WM are built based on fuzzy minimum distance classification, which results in fuzzy volumes of brain tissues (Aubert-Broche et al., 2006).

#### 2.3.2 Freesurfer: surface model-based segmentation using an adult atlas

The Freesurfer version 5.3.0 computes the tissues volumes by applying a surface model-based segmentation. After affine registration towards the MNI305 atlas that is built from 305 T1-weighted images of healthy young adults (Collins et al., 1994) and bias field correction, the image is skull-stripped with a deformable surface template model. Based on the skull-stripped image, brain tissue segmentations are obtained with both the surface-based stream (Dale et al., 1999) and the volume-based stream (Fischl et al., 2002). In the surface-based stream, starting from a surface brain template, the white matter outer surface is first delineated based on the intensity and neighbor constraints and is then refined based on intensity gradients between GM and WM. The pial surface is afterwards defined by pushing gradually the white matter outer surface to the boundaries between GM and CSF, also based on intensity gradients between both tissues. In the volume-based (subcortical) stream, tissue labels of the MNI305 atlas are propagated to the subject image to assign a brain tissue to each voxel. This stream is mainly used to correct the segmentation in the subcortical areas. The volumes are computed from both surface-based volume and voxels counts. To have the same definitions of GM and WM with methods described previously, GM included the cortical, subcortical and cerebellum gray matter and WM includes cortical and cerebellum white matter, and also the brainstem. For a fair comparison with the other methods, default parameters were used and the images were not corrected by means of manual editing.

### 2.4 Methods evaluation

Segmentation performance of each method was assessed based on three criteria: the reproducibility, the robustness to low image quality and the segmentation accuracy. The evaluation measures obtained with child**metrix** were compared to those obtained with the two state-of-the-art techniques, ico**brain** and Freesurfer. Finally, the impact of the method performance on further statistical analyses is also assessed by comparing tissue volume distributions between children with and without dyslexia estimated by the three automated methods.

The statistical analyses were performed using R packages (R Core Team, 2013). As the performance measures were not normally distributed (assessed with Shapiro-Wilk test), differences between automated methods were assessed using pairwise Wilcoxon signed rank tests, with *p*-value (*p*) under 0.05 considered as significant. In order to compare the correlations, a correlation difference test was performed using Fisher r-to-z transformation, with p-value under 0.05 considered as significantly different. All *p*-values were corrected for multiple comparison by means of Holm correction.

#### 2.4.1 Reproducibility

The reproducibility is the ability of the method of obtaining the same results when taking several measurements under the same conditions. The reproducibility was measured based on the segmentations and volumes extracted from test-retest scans of the NKI dataset. Two criteria were then used to assess the reproducibility:

• **Overlap of brain segmentations**: an affine registration step is performed between test-retest scans, to align tissue segmentations. The Dice overlap coefficient (Dice, 1945) between segmentations (*O*) is defined as the intersection of voxels assigned as tissue (*v*) for the first scan (*s*_1_) and for the second scan (*s*_2_) divided by the mean number of voxels assigned as tissue.

$$O\left(s_{1},s_{2}\right)=\frac{\left(v\left(s_{1}\right)\cap v\left(s_{2}\right)\right)}{\left(v\left(s_{1}\right)+v\left(s_{2}\right)\right)/2}\times 100$$

For methods returning a probabilistic segmentation (child**metrix** and ico**brain**), the voxel is assigned as tissue when the probability in that voxel is equal to or above 0.5.

• **Volume percent difference**: for each subject, the volumes were computed for the two test-retest scans. The volume percent difference is defined as the absolute difference of the volumes (V) divided by the mean volume between test-retest scans.

$$\mathrm{\Delta V}\left(s_{1},s_{2}\right)=\frac{\left|\left(V\left(s_{1}\right)-V\left(s_{2}\right)\right)\right|}{\left(V\left(s_{1}\right)+V\left(s_{2}\right)\right)/2}\times 100$$

A high overlap in segmentation (close to 100%) and small volume difference (close to zero) correspond to high reproducibility.

#### 2.4.2 Robustness to lower image quality

Robustness to lower image quality is the ability of the method to be unbiased by noise and artifacts, which are often present in pediatric data. The robustness was measured by using the Spearman correlation between the volumes computed by the automated methods and the quality measures (i.e. Blumenthal's motion, SNR and CNR, described in Section 2.1.2) on the DYSCO dataset. A high correlation corresponds to a low robustness to low image quality, as the results depend on the presence of noise or artifacts.

#### 2.4.3 Segmentation accuracy

The segmentation accuracy is the ability of the method of providing segmentation that is close to the ground truth. The accuracy was then measured based on the Dice overlap coefficient (*O*) between the automated segmentation (S_a_) and the manual segmentation (S_m_) of one human rater, defined as the following:

$$O\left(S_{a},S_{m}\right)=\frac{\left(S_{a}\cap S_{m}\right)}{\left(S_{a}+S_{m}\right)/2}\times 100$$

Two subjects of the DYSCO project were manually segmented for GM and WM. The two subjects were selected to represent a scan with low image quality (SNR = 242, CNR = 110, moderate motion) and a scan with good quality image (SNR = 700, CNR = 314, no motion in Blumenthal's rating). The manual segmentation was performed by correcting the automated segmentation obtained with Freesurfer in 3Dslicer, a software platform for medical image processing and visualization (Fedorov et al., 2012). The reliability of the manual segmentation was assessed by measuring inter-rater reliability, which was between 85.1% and 91.3% overlap with four other raters who performed the segmentation on one of the same two subjects.

#### 2.4.4 Ability to capture group differences

In order to evaluate the impact of the method on further clinical analyses, we performed a power analysis to assess the ability to capture differences between clinical groups for the different methods. The power analysis estimated the sample size based on the effect size between a group of children with typical reading skills (21 subjects, after removing scans with severe motion) and a group of children with dyslexia (13 subjects), both part of the DYSCO dataset. The tissue volume distributions for each group were normally distributed. The size effect of difference was measured with the Cohen's d, which is defined as the difference between two means divided by the pooled standard deviation (Cohen, 1977). The d value corresponds to a “very small” effect size for a value around 0.1, “small” for a value around 0.2, “medium” for a value around 0.5, “large” for a value around 0.8, “very large” for a value around 1.2 and “huge” for a value around 2 (Sawilowsky, 2009). Based on the Cohen's d, we computed the required sample size in order to assess a significant effect with a power of 0.8 and a significance of 0.05, using the software tool G*Power version 3.1.9.2 (Faul et al., 2007).

## 3 Results

### 3.1 Quality assessment

The quality assessment for the scans of children at kindergarten from the DYSCO project is summarized in Table 1. It shows the proportion of scans classified in each category (none, mild, moderate and severe), together with mean SNR and CNR of each category. The quantitative measures confirm the qualitative measure based on visual inspection since images with little motion have on average higher SNR and CNR, and vice versa. Scans with severe motion (17 out of the 72 scans) were excluded from the analyses, as those scans are difficult to analyze by both manual and automated segmentation. Hence, 55 out of the 72 scans were included in the statistical analyses.

**Table 1:** Motion rating of scans from children in kindergarten (*n* = 72) in longitudinal DYSCO MRI-dataset with corresponding mean SNR and CNR.

Blumenthal's motion rating	Proportion of the dataset	Mean SNR	Mean CNR
(1) None	33.4%	1690.3	868.0
(2) Mild	23.6%	535.8	251.1
(3) Moderate	19.4%	167.4	82.3
(4) Severe	23.6%	145.3	58.8

Concerning the NKI dataset, the quality of both test-retest scans was also assessed following Blumenthal's motion rating. Out of the 26 scans, there was 1 scan with no motion (SNR = 301.5, CNR = 65.7), 9 with mild motion (mean SNR = 285.4, mean CNR = 63.0), 13 with moderate motion (mean SNR = 252.9, mean CNR = 57.8) and 3 with severe motion (mean SNR = 176.0, mean CNR = 40.8). The three subjects with severe motion in one of the test-retest scans were excluded from the analysis. Hence, 10 pairs of test-retest scans were included in the statistical analyses.

### 3.2 Assessment of reproducibility (NKI dataset)

**Fig. 3:**
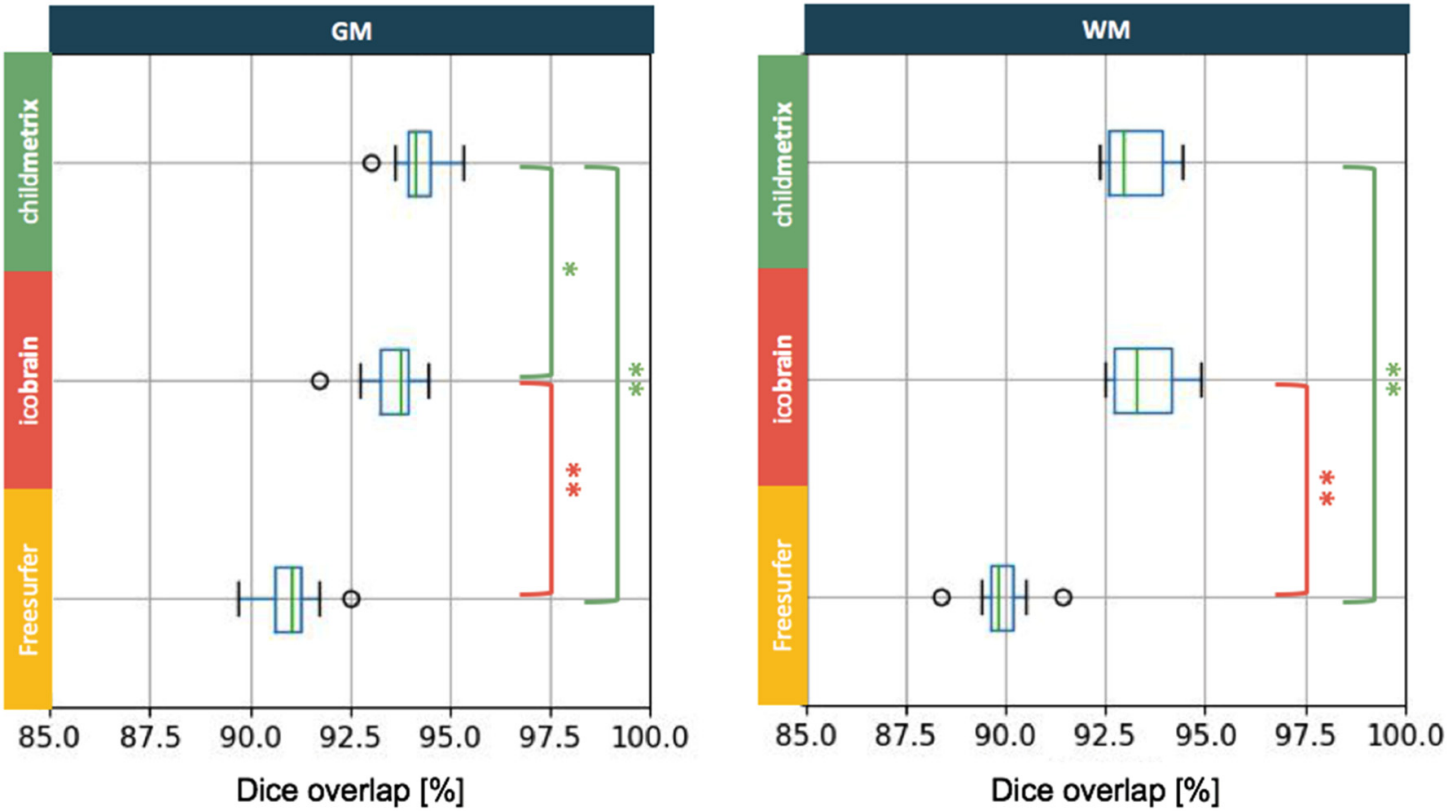
Comparison between the three segmentation methods based on Dice overlap coefficient between test-retest scans (with * corresponding to *p*-value <0.05 and ** to *p*-value <0.01 for pairwise Wilcoxon signed rank test with Holm correction for multiple comparison).

Assessed on the NKI dataset (10 subjects), we evaluated the reproducibility based on the Dice overlap coefficient and the percent volume difference between test and retest scans. A high reproducibility corresponds to a Dice overlap close to 100% and a percent volume difference close to zero. The results based on the Dice overlap showed that child**metrix** (using the NIHPD 4–8 atlas) was more reproducible than the two other methods, especially for GM segmentation (see Fig. 3). For GM, the overlap was the highest on average for child**metrix**, with a value of 94.21%, followed by ico**brain** with a value of 93.53%, and then Freesurfer with a value of 90.98%. There was a significant difference when child**metrix** was compared with ico**brain** (*p* < 0.05) and with Freesurfer (*p* < 0.01), and there was also a significant difference between ico**brain** and Freesurfer (*p* < 0.01). Hence, the differences in overlap were driven by the choice of atlas and by the segmentation algorithm. For WM, the highest average overlap was reached by ico**brain** with a value of 93.45%, followed by child**metrix** with a value of 93.25% and then Freesurfer with a value of 89.89%. Results were not significantly different between child**metrix** and ico**brain**, but they were different between child**metrix** and Freesurfer (*p* < 0.01) and between ico**brain** and Freesurfer (*p* < 0.01). Hence, the differences in WM segmentation overlap were mainly driven by the segmentation algorithm, with the highest overlap for probabilistic model-based segmentation.

**Fig. 4:**
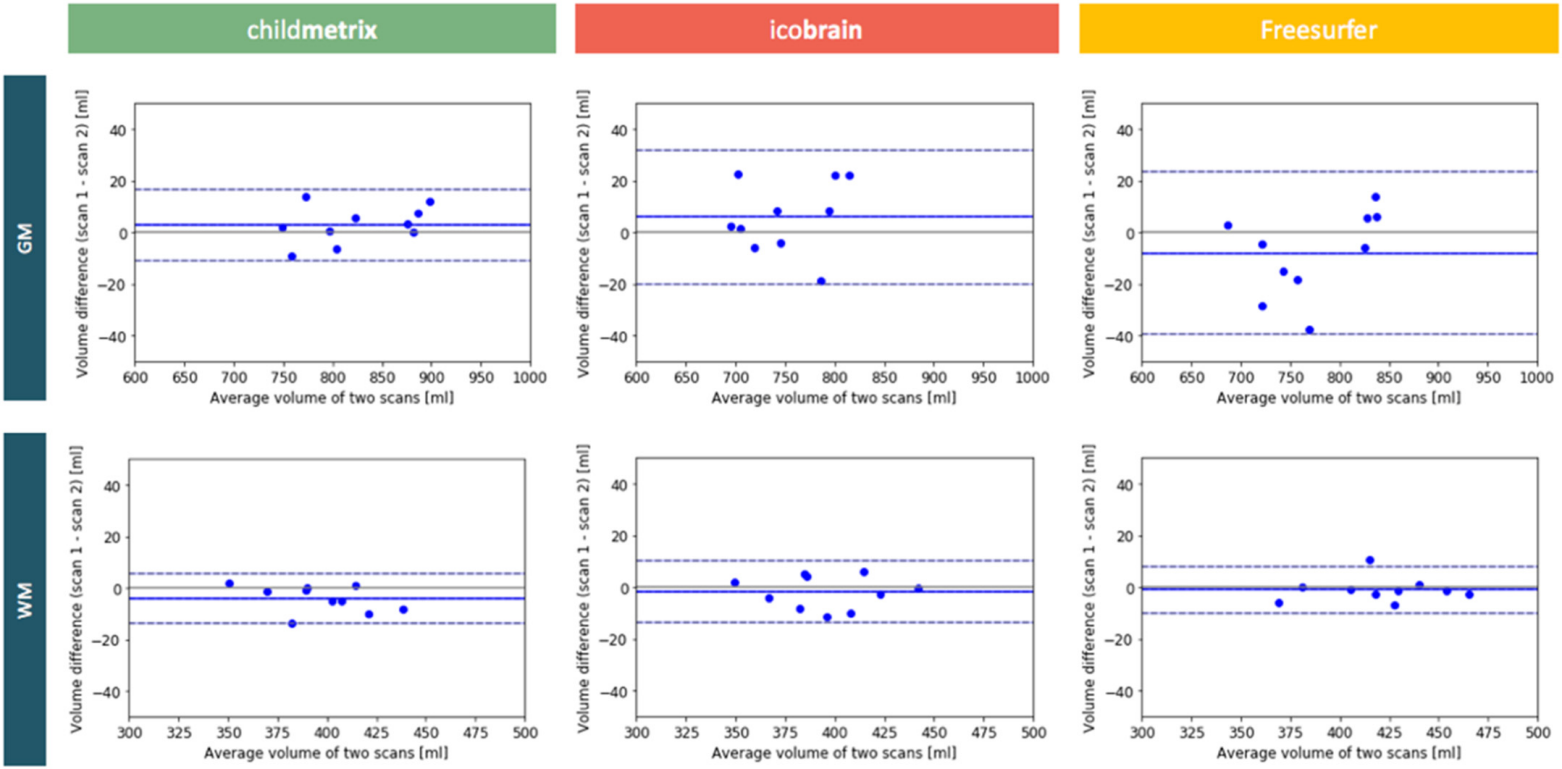
Bland-Altman plot for agreement between test-retest scans based on GM and WM volume computed by each segmentation method.

To visualize the agreement between volume estimation in the test and retest scans, Bland-Altman plots for GM and WM volume computed by each method are illustrated in Fig. 4. The average percent volume differences for GM and WM were 0.37% and 0.58% respectively for child**metrix**, 0.75% and 0.68% respectively for ico**brain** and, 0.91% and 0.40% respectively for Freesurfer, with no significant difference between the three methods.

### 3.3 Assessment of robustness to low image quality (DYSCO dataset)

**Fig. 5:**
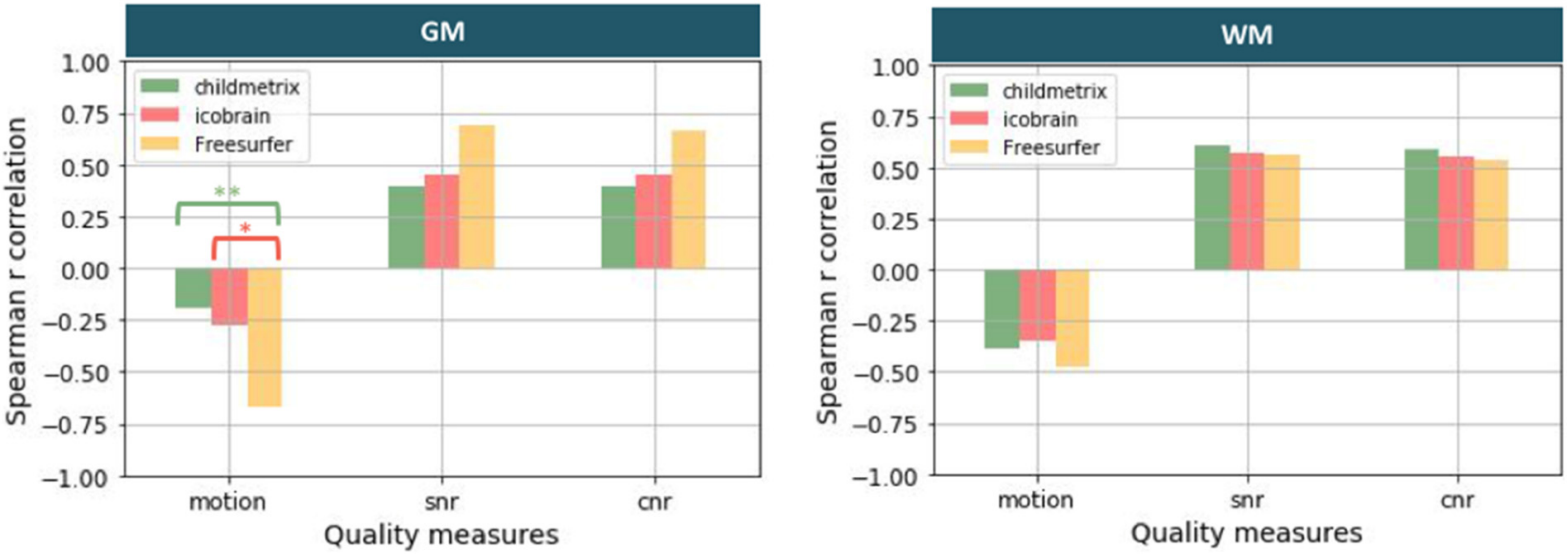
Spearman correlation coefficient between tissue volume and quality measure (with motion: Blumenthal's motion rating, snr: signal-to-noise ratio, cnr: contrast-to-noise ratio, and * corresponding to p-value <0.05 and ** p-value <0.01 for correlation difference test with Holm correction for multiple comparison).

On the DYSCO dataset (55 subjects), we evaluated the robustness to low image quality by comparing correlations between tissue volumes and quality measures for the different methods (see Fig. 5). A correlation coefficient closer to zero corresponds to a higher robustness of volumetric results to poor image quality. Concerning the robustness to head motion, correlations were not significant between Blumenthal's motion rating and GM volumes estimated by child**metrix** (*r* = −0.19) and ico**brain** (*r* = −0.28), conversely to Freesurfer (*r* = −0.67, *p* < 0.01). WM volumes estimated by all methods were significantly correlated to head motion (*p*_*s*_ < 0.05). The correlations between Blumenthal's motion rating and GM volumes were significantly lower for child**metrix** relative to Freesurfer and for ico**brain** relative to Freesurfer. No difference in correlations with WM volumes was observed between all methods. Hence, the differences in correlation with GM volumes are mainly driven by the segmentation algorithm, with a higher robustness for the probabilistic approach. Concerning the robustness to noise, correlations were all significant between SNR (or CNR) and tissue volumes (*p* < 0.05), and correlations were not significantly different when comparing the three automated methods. These results showed that all methods are sensitive to noise.

### 3.4 Assessment of segmentation accuracy (DYSCO dataset)

The segmentation accuracy was assessed on two subjects from the DYSCO dataset (one with a T1-scan of good quality and the other with a scan of low quality), based on the Dice overlap between the manual segmentation and the automated segmentation. The results showed that the highest overlap with manual segmentation was obtained with child**metrix** for both GM and WM when compared to the two adult-based method, ico**brain** and Freesurfer (see Table 2). When comparing the Dice overlap coefficients between the scan of good quality and the scan of low quality, the accuracy was similar when the segmentation was performed using child**metrix** and ico**brain**. For Freesurfer, the results were similar for WM but for GM, there was a drop of about 12% in the Dice overlap coefficient when the image quality is low compared to the scan of good quality.

**Table 2:** Agreement measure (dice overlap coefficient) between manual segmentation and automated methods.

Automatic method	Dice overlap coefficient (with manual segmentation)			
	Scan of good quality		Scan of low quality	
	GM	WM	GM	WM
child**metrix**	82.05%	82.65%	83.74%	83.66%
ico**brain**	77.77%	79.55%	75.96%	80.47%
Freesurfer	80.69%	79.69%	68.77%	78.01%

**Fig. 6:**
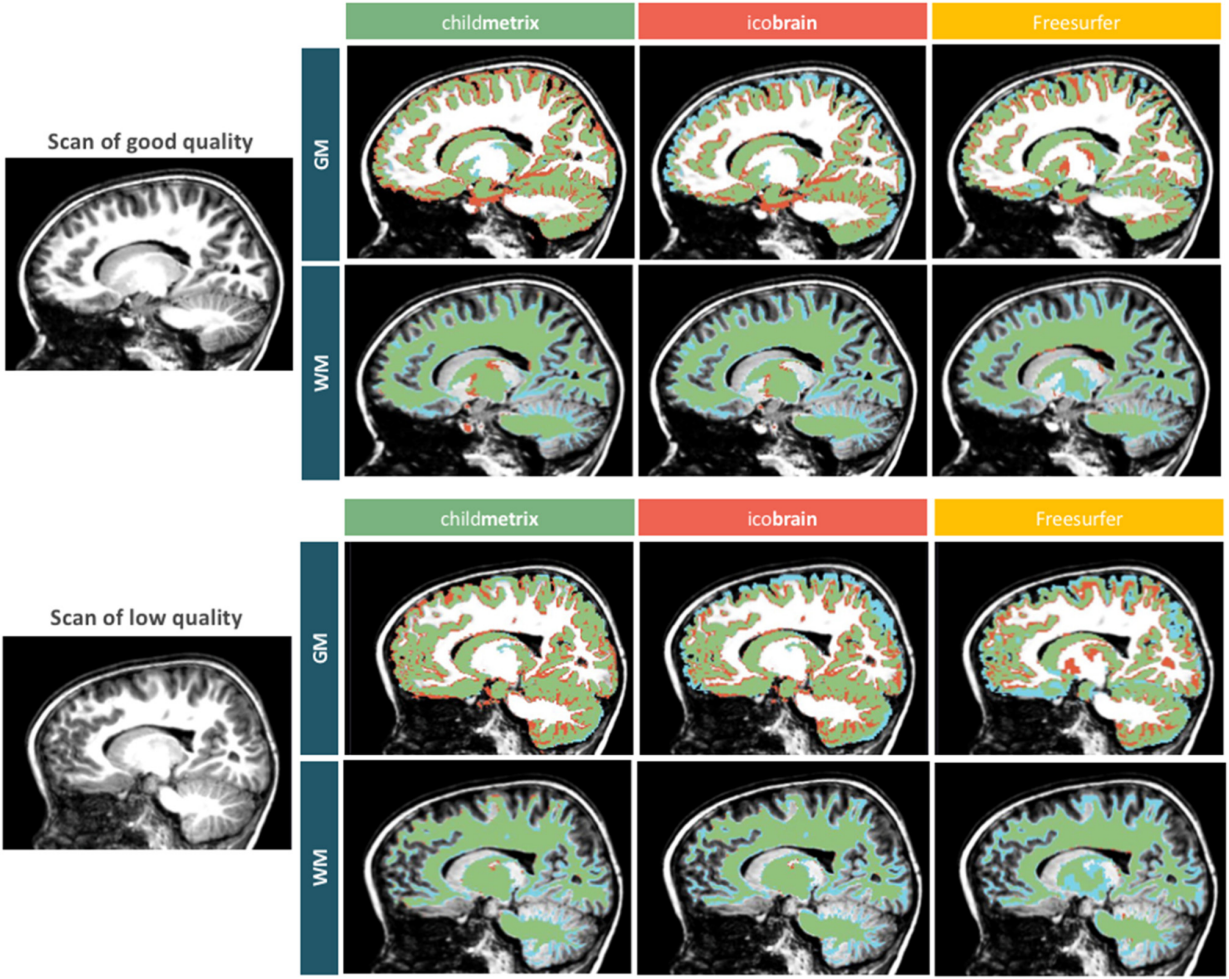
GM and WM segmentation of a pediatric scan of good quality (SNR = 700, CNR = 314, no motion) on the top and a scan of low quality (SNR = 242, CNR = 110, moderate motion) below, performed for each automated method (blue: manual segmentation, red: automated segmentation and green: agreement).

The agreement between manual segmentation and automated segmentation is illustrated in Fig. 6. Systematic errors made by the automated methods were assessed by visual inspection. These errors were observed for all methods at the boundary between GM and WM, which might be due to the partial volume effect or the level window used during the manual delineation that changes the threshold on the intensity in order to distinct both tissues. The contour of cortical GM was relatively well delineated by child**metrix** and Freesurfer. This was not the case for ico**brain** that provided an under-segmented cortical GM. This error seems to come from a poor delineation of the brain mask by ico**brain** that impacts later the delineation of GM and WM. The contour of cortical WM was better defined by child**metrix**, which was able to capture smaller structures than the two other methods. The subcortical areas were areas that were hard to accurately delineate for the three automated methods, especially in the thalamus and the globus pallidus. The cerebellum was in general well delineated by the three methods, but the smaller structures in cerebellum could not be well segmented. In regards to the image quality, the results from Table 2 demonstrated the low performance of Freesurfer and the stability of ico**brain** and child**metrix** for GM segmentation of low quality images, confirming the results obtained during the robustness assessment (see Section 2.4.2).

### 3.5 Methods comparison in group comparison (DYSCO dataset)

The results for group comparison between children with dyslexia (*n* = 13) and children with typical reading skills and no family risk (*n* = 21) are shown for GM and WM volume in Table 3. The Cohen's d values were higher for child**metrix** when compared with ico**brain** and Freesurfer. The difference of effect size is particularly observed for GM volume for which the effect size was considered as “large” for child**metrix** and for ico**brain**, and “medium” Freesurfer according to Cohen's d. The required sample size per group in order to measure a significant effect with a power of 0.8 and a significance of 0.05 was the smallest for child**metrix**. Only half the number of subjects is required for child**metrix** when compared with Freesurfer in order to find significant differences in GM volumes. For WM, one third additional subjects would have been required for Freesurfer when compared to child**metrix** in order to find significant differences.

**Table 3:** Group comparison of total brain volume between children with typical reading skills (n = 21) and with dyslexia (=13).

Methods	Mean (standard deviation)		Cohen d	Required sample size per group
	Typical [ml]	Dyslexic [ml]		(Power:0.8 and significance: 0.05)
Gray matter volumes				
child**metrix**	846.0 (60.5)	802.4 (62.1)	0.76	23
ico**brain**	709.6 (56.4)	672.8 (54.1)	0.70	26
Freesurfer	674.7 (85.6)	640.7 (51.7)	0.48	55

White matter volumes				
child**metrix**	402.5 (36.4)	379.9 (32.2)	0.68	28
ico**brain**	376.9 (35.7)	356.4 (29.9)	0.65	30
Freesurfer	400.5 (52.4)	373.8 (43.2)	0.58	38

## 4 Discussion

In this paper, we introduced and validated child**metrix**, a fully automated tool for volumetric analyses of pediatric brain MRI. The results demonstrated that child**metrix** provides better automated segmentations of GM and WM compared with ico**brain** and Freesurfer with regards to reproducibility and robustness to head motion. The child-adjusted method also seems to improve the segmentation, compared to the adult-based methods. These improvements are important as they might have an impact on further analyses, such as group comparisons.

Reproducibility is an important feature for automated methods, as good reproducibility can provide a benefit of applying automated methods compared to manual segmentation that might be subject to low reproducibility. Manual segmentation of structures in the brain could achieve intra- and inter-rater variability above 10% (Ashton et al., 2003; Entis et al., 2012). Compared to the manual reliability assessed in this study (between 85.1% and 91.3% inter-rater overlap on the same scan), the average Dice overlap between the test and retest scans was above this range for ico**brain** and child**metrix** (>92% overlap on average) and for Freesurfer, the average Dice overlap was in the range of the inter-rater reliability. According to the results based on the Dice overlap between test and retest scans, the reproducibility in GM and WM segmentation is mainly driven by the segmentation algorithm, with better results obtained with the probabilistic model-based segmentation compared with the surface-based segmentation. Using an age-specific atlas has also lead to higher reproducibility compared to the standard adult atlases, although this was only the case for GM. The reproducibility results based on the volume difference between test and retest scans did not show any difference between methods. This contradictory result between Dice overlap and volume difference might be explained by the fact that the three automated methods can provide similar values of tissue volumes for the test and retest scans, but the spatial location of the tissue (i.e., voxel-by-voxel overlap) could be better reproduced by child**metrix** and ico**brain**. A low reproducibility is particularly problematic for the study of brain development and neurodevelopment disorder, as changes over time and differences between groups might be introduced or hidden by intrinsic variability of the method. To be appropriate for these applications, the percent volume difference should be below the relative volume change per year, which is about 1% for WM and 0.5% for GM in children below 10 years old (Hedman et al., 2012). The three automated methods could achieve an average percent volume difference below 1% for WM, but only child**metrix** could achieve an average percent volume difference below 0.5% for GM. This means that child**metrix** would better capture subtle changes in GM than the two adult-based methods.

Low image quality is a critical issue in the analysis of pediatric brain data. Particularly, head motion is inherent to pediatric studies as children are often less compliant to stay still in the scanner compared to adults (Theys et al., 2014). As a consequence, a significant proportion of scans would be excluded from the analysis, because the low image quality renders them unusable. In this study, 23.6% of the dataset had to be excluded of the analysis because of severe motion visible in the scan. Although mild and moderate motion have been shown to lead to bias in the measurement of brain structure properties (Alexander-Bloch et al., 2016; Blumenthal et al., 2002; Reuter et al., 2015), excluding these scans would have led us to remove >50% of the dataset, which would considerably reduce the statistical power. In this study, we showed that GM volume estimation by child**metrix** and ico**brain** was not significantly correlated with motion (from none to moderate), whereas Freesurfer was significantly correlated. In addition, other quality measures, such as SNR and CNR, also showed a weaker correlation with tissue volumes estimated by child**metrix** and ico**brain** than by Freesurfer, but the correlations were not significantly different between methods. These results are promising because scans with mild and moderate motion processed by childmetrix and ico**brain** could be included in the statistical analysis since the volume measures are less biased by motion. The robustness to motion for child**metrix** and ico**brain** might come from the probabilistic model for which spatial constraints enable to maintain a plausible segmentation even in the presence of motion artifacts. In contrast, Freesurfer uses a surface model-based segmentation method, which had been showed to be slightly more sensitive to motion than other probabilistic methods, such as SPM and SIENA (Reuter et al., 2012). WM volume estimation seems more sensitive to low image quality since it was significantly correlated to motion, SNR and CNR for all methods, yet here no difference between the methods was found. The significant correlation with SNR and CNR could be related to the dependency on image intensity that is used by both types of segmentation algorithm (probabilistic and surface based segmentation) in order to segment GM and WM. Indeed, the distinct peaks of GM and WM in the intensity distribution might be merged when SNR and CNR are low, which hinders the distinction between both tissues (Despotović et al., 2015). This effect is enhanced in the presence of motion artifacts, for which the detection of WM is even more hindered. Diffusion MRI might be a complementary modality to use in order to measure adequately structural properties of WM, but child-adapted methods based on this modality should be further investigated in pediatric populations.

The segmentation accuracy is essential in order to obtain measures that are close to reality and to observe genuine differences over time and between groups, which might be subtle when studying neurodevelopmental disorders (Ramus et al., 2017; Schumann et al., 2010; Valera et al., 2007). It is therefore crucial to know whether the automated segmentations also correspond to reality. In this study, the accuracy was assessed based on the overlap between the automated segmentations and the manual segmentation that was used as the gold standard. Our results suggest that methods based on an age-specific atlas, whether it is independent or study-specific (see Supplementary material), provide more accurate segmentation than methods based on an adult atlas. In line with studies on adapted segmentation tools for infant brains (Murgasova et al., 2007; Shi et al., 2011), improved accuracy were obtained when using an age-specific atlas in older children (here, 5–8 years old), which supports the need of using age-specific atlases for this age group. Similar validations should also be conducted in children older than 6 years old in order to determine to which extent age-specific atlases can be useful. In regards to WM segmentation, conversely to GM, using an age-specific atlas on children of 5–8 years old did not show improved performance compared to using an adult atlas. A possible explanation is that the structural organization of WM in 5 years old starts to be similar to the organization within the adult brain, with the developmental trajectory relatively consistent across the major lobes and with a smaller rate of changes, while the developmental trajectory of GM follows an inverted U-shaped curve, with a maximum reached during childhood (around the same period as the studied subjects) and with regionally specific rate of changes (Aubert-Broche et al., 2013; Brain Development Cooperative Group, 2012; Courchesne et al., 2000; Hedman et al., 2012; Mills et al., 2016; Mills and Tamnes, 2014). Therefore, it seems that the use of an age-specific atlas is less needed in order to study the WM from 5 years of age onwards.

A limitation of our study was that the accuracy was quantitatively assessed on only two subjects since manual segmentation is time-consuming (here, around 100 h for GM and WM segmentation per subject). As a consequence, we could not evaluate the accuracy with statistical tests and neither relate it to the four categories of motion rating. However, the two subjects were specifically chosen to be representative of the cohort, considering one image with good quality (corresponding to no motion) and one with poor quality (corresponding to moderate motion), assuming that image of medium quality (with mild motion) would lead to intermediate results. With the same pattern of errors observed in other subjects by visual inspection, we expect that our accuracy results are generalizable to the whole dataset, and other pediatric samples. Another limitation to consider is that the manual segmentation consisted of correcting the segmentation obtained first with Freesurfer, hence, a bias towards Freesurfer was expected. However, despite the potential bias, the accuracy results were still in favor of child**metrix** showing higher overlap with manual segmentation for the two subjects. Similar accuracy was obtained with child**metrix** and ico**brain** on images of low quality when compared with image of good quality, which was not the case for Freesurfer for which the accuracy dropped drastically in presence of moderate motion. These results showed again that the segmentation algorithm mainly plays a role in the robustness to low image quality, which impacts later on the tissue delineation.

The impact of the segmentation method on brain volumetric analyses was assessed by comparing group differences between typical reading and dyslexic children estimated by each method. In a recent review paper, it has been shown that the effect size of whole brain group differences is highly variable between studies investigating dyslexia (Ramus et al., 2017). This might be due to differences in sample characteristics but as our results show it might also be due to differences in the methods used to analyze whole brain volumetric differences. More specifically, our results showed that the improvement brought by child**metrix** in the segmentation impacts the detection of subtle volume difference between children with dyslexia and children with typical reading skills, reflected by higher effect sizes. This implies that child**metrix** requires a smaller sample size (up to half the sample size) in order to detect a significant effect compared with the two adult-based methods. A reduced sample size that enables a high statistical power is particularly relevant for pediatric studies, and even more for studies investigating neurodevelopmental disorders, for which the recruitment of participants is more difficult and the exclusion of a significant proportion of dataset is more likely to occur due to low image quality.

With the evaluation performed on children of 5–8 years old, we demonstrated the need of using an adapted tool for this age-group, which stands in contrast to the general belief that well-established methods developed for adults are suitable for pediatric data from 5 years of age onwards. In this paper, we compared results obtained with an age-specific atlas and with an adult atlas using the same EM segmentation. The same comparison could have been done for methods based on surface models such as Freesurfer, but to our best knowledge, age-specific surface templates are not yet available for the targeted age range (here, 5–8 years old). In this study, we did not assess the performance of Freesurfer using an age-specific atlas because no pediatric surface brain atlas was available for use in Freesurfer. Still, we might infer from the comparison between Freesurfer and ico**brain** that Freesurfer will perform worse than child**metrix** in pediatric images of low quality as the surface-based algorithm is sensitive to motion artifacts, low SNR and low CNR. The need for tools adapted to pediatric populations can be generalized to other segmentation methods. For example, studies based on machine learning methods have shown similar observations for neonatal and adult brains by using training data that are representative of the targeted populations (Moeskops et al., 2016, Moeskops et al., 2015; Wang et al., 2015). Similarly to what we did in this study, an extensive evaluation on different age-groups in the human lifespan can be performed with these different segmentation algorithms using age-specific data as reference in order to validate the need of using them in general.

As a tool to be further made publicly available, child**metrix** still needs to be improved in order to help researchers answering questions related to children's brain and neurodevelopmental disorders, but also to its development. As GM and WM volumes are interesting measures to assess some neurodevelopmental disorders, volume measurement of specific regions-of-interest enables to better assess a particular disorder (Levman and Takahashi, 2015). Therefore, additional functionalities should be developed and adapted to pediatric data, such as pipelines for longitudinal and region-specific processing which are already included in the two standard software tools, ico**brain** and Freesurfer.

## 5 Conclusion

In conclusion, it has been demonstrated that the proposed automatic tool for whole brain volumetric analysis in pediatric data, chil**metrix**, provides more reproducible and robust results than Freesurfer. The results tended to show a higher segmentation accuracy when using the child-adjusted method compared to using the adult-based methods, but this should be validated on more subjects. In this paper, we demonstrated on real data the importance of having a tool that is suitable for children between 5 and 8 years old, an age-group that has been neglected with the expectation that well-established methods made for adults would be suitable. Similar experiments should be investigated more in depth for even older children. Once further developed and validated, we believe that child**metrix** would provide reliable and more sensitive measures for the examination of children's brain and its development, particularly in order to monitor and help children affected with neurodevelopmental disorders.

## Appendix A Supplementary data

Supplementary material Image 1
