# Supplementary material for: Evaluation of methods for volumetric analysis of pediatric brain data: The childmetrix pipeline versus adult-based approaches
Source: Neuroimage Clin. 2018 May 23;19:734–44. doi: 10.1016/j.nicl.2018.05.030 (PMC6040578; doi:10.1016/j.nicl.2018.05.030)
Supplement: Supplementary file 1 — Supplementary material [file mmc1.docx]

Evaluation of methods for volumetric analysis of pediatric brain data: the child**metrix** pipeline versus adult-based approaches.

Thanh Vân Phan^a,b^, Diana M. Sima^a^, Caroline Beelen^c^, Jolijn Vanderauwera^b,c^, Dirk Smeets^a^, Maaike Vandermosten^b^

^a^ ico**metrix**, Research and Development, Leuven, Belgium

^b^ Experimental Oto-rhino-laryngology, Department Neurosciences, KU Leuven, Leuven, Belgium

^c^ Parenting and Special Education Research Unit, Faculty of Psychology and Educational Science, KU Leuven, Leuven, Belgium

**Corresponding author:** Thanh Vân Phan, [van.phan@icometrix.com](mailto:thanhvan.phan@kuleuven.be)

**Permanent address**: ico**metrix**, Department of Research and Development, Kolonel Begaultlaan 1b/12, 3012 Leuven, Belgium

# **Supplementary Material**

# **Comparison between study-specific atlas and population-based atlas**

In order to assess the potential added-value of a study-specific atlas above an independent age-specific atlas, we created the **DYSCO 5-6 atlas** and used it as reference in the segmentation pipeline when it was applied on data of the DYSCO project. The study-specific atlas would have the advantage of being more similar to the images from the same study (similar contrast due to same protocol conditions, similar populations, etc.), which might lead to less error of registration.

## **Pipeline to build the study-specific atlas**

The template creation pipeline of child**metrix** (illustrated in **Fig. *S.*1**) follows a different strategy compared to the NIHPD 4-8 atlas and consists of the five following processing steps. As a first step, subjects are selected to be part of the average head template. In the second step, all selected images are aligned in MNI space with an affine registration, using the NiftyReg method that is based on Trimmed Least Square scheme and a block matching approach (Ourselin et al., 2000). In the third step, the non-rigid transformations (to warp the image of one subject towards the images of other subjects) are computed from non-rigid registration, using Free-Form deformations of NiftyReg (Modat et al., 2010) and are then averaged for each subject. In the fourth step, the images are warped with the corresponding average non-rigid transformation computed at the previous step in order to obtain the average shape. Finally, the image intensity is averaged across all warped images in the fifth step. The final result corresponds to the brain template in MNI space, which summarizes the brain anatomy of the selected subjects. In this study. the created template aimed at representing the average brain structures of the control and dyslexic groups of children at kindergarten (5-6 years old) studied in the DYSCO project. To build the template, twenty-five out of the 72 (35%) T1-weighted images from the DYSCO were selected based on the image quality, which had to be relatively good (with none or mild motion and high SNR/CNR) and were then corrected for bias field with the N4 algorithm of ANTS (Tustison et al., 2010). Seventeen of these children developed typical reading skills whereas 8 children developed dyslexia. Once the brain template was created, the brain mask was manually delineated to fit the brain template and the tissue probability maps were the same as the NIHPD 4-8 atlas with no warping required.


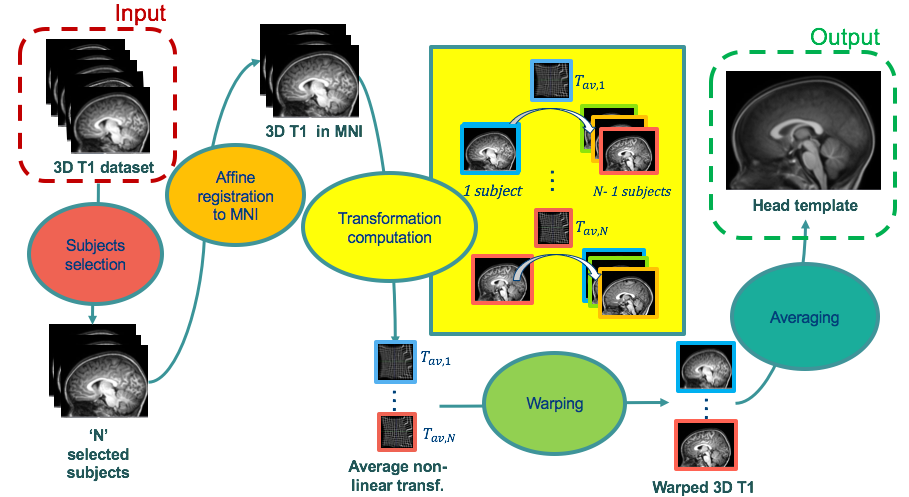


**Fig. S.1.** Scheme of creating a study-specific brain template in child**metrix.**

## **Comparison on the brainmasking**

Because the brain mask is specific to the DYSCO 5-6 atlas (manually delineated on the head template), we expect a difference in the brain masking results (outcome of the skull stripping steps). The assessment was done on the 72 subjects from the DYSCO dataset. The two atlases were compared based on the Dice coefficient measuring the overlap between the mask obtained with the NIHPD 4-8 atlas and the mask obtained with the DYSCO 5-6 atlas on the same subject. The results showed a mean Dice overlap of 96,55% with a standard deviation of 0.87%. Based on visual inspection, these small differences were observed in areas anterior to the brainstem, posterior to the cerebellum, anterior to the frontal lobe and posterior to the parietal and occipital lobes, as illustrated in the example (see **Fig. *S.*2**).


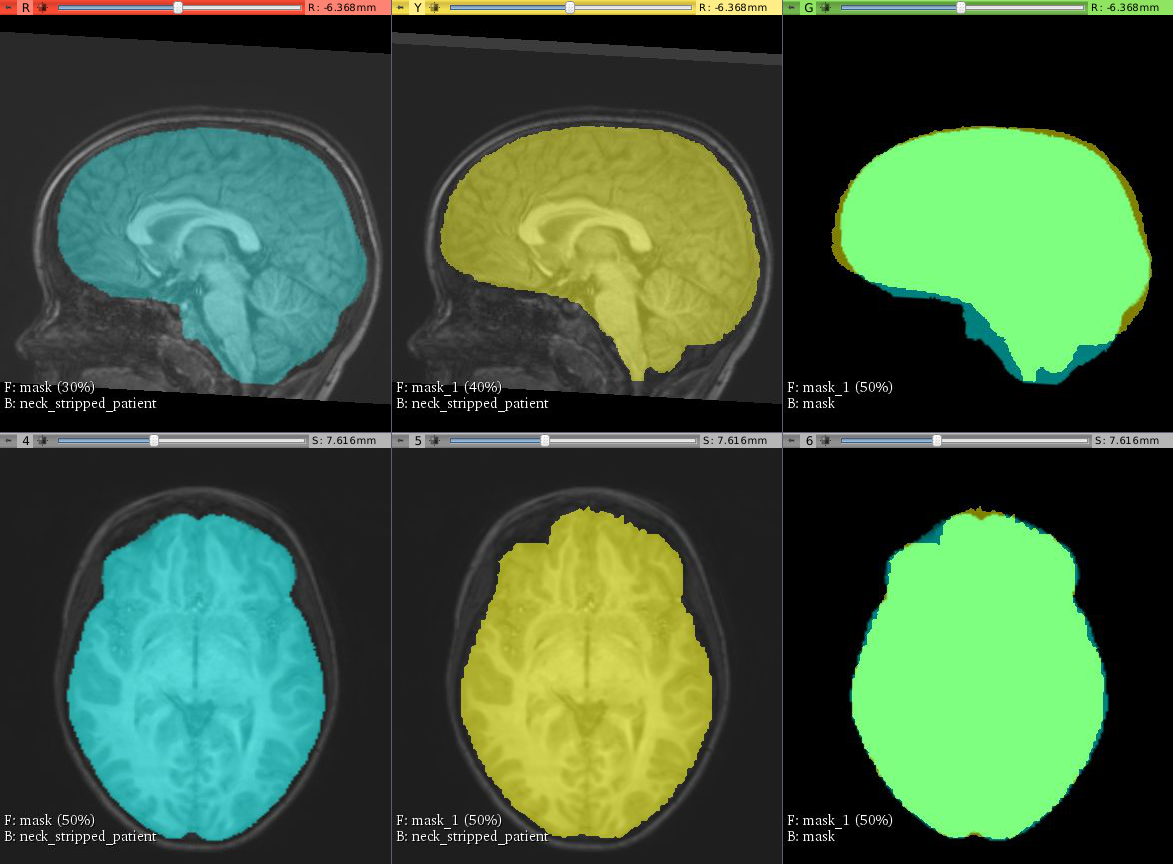


**Fig. S.2.** Brain mask obtained with the NIHPD 4-8 (blue) and with the DYSCO 5-6 (yellow) and their overlap (green). The two brain masks are overall overlapping, with small differences in areas anterior to the brainstem, posterior to the cerebellum, anterior to the frontal lobe and posterior to the parietal and occipital lobes.

## **Comparison on the atlas registration**

The atlas registration is based on the brain template and therefore, we also expect also differences at this step as the two brain templates are different. The assessment was done on the 72 subjects from the DYSCO dataset. The results obtained with the two atlases were compared based on the normalized mutual information (NMI) measuring the similarity between the images of the subject and the template registered to the subject (the closer is NMI to one, the higher is the similarity). The results showed a mean NMI of 0.656 (and standard deviation of 0.018) obtained with the NIHPD 4-8 atlas and a mean NMI of 0.665 (and standard deviation of 0.005) obtained with the DYSCO 5-6 atlas. Although it is not the case of for all images, the DYSCO 5-6 atlas can be considered a bit more similar to the images from DYSCO dataset compared NIHPD 5-8, as illustrated by a smaller variance and a higher median value (see **Fig. *S.*3**).


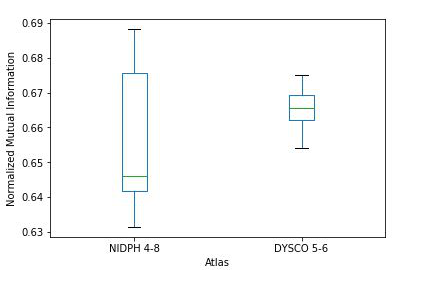


**Fig. S.3.** Normalized Mutual information measuring the similarity between the images and the atlas registered to the images from DYSCO dataset (72 subjects). The results are showing that the DYSCO 5-6 atlas is in average a bit more similar to the images from DYSCO dataset, although it is not the case for all images.

By visual inspection (as the example illustrated in **Fig. *S.*4**), we could observe that the big WM structures fit well the image of the subject, but it is not the case for the small structures that presents more anatomical variability. As the NIHPD 4-8 atlas is a sharp atlas, the WM structures of the atlas were not well matched to the WM structures of the subject, while for the smooth DYSCO 5-6 atlas, these structures corresponded to very blurred areas in the atlas.


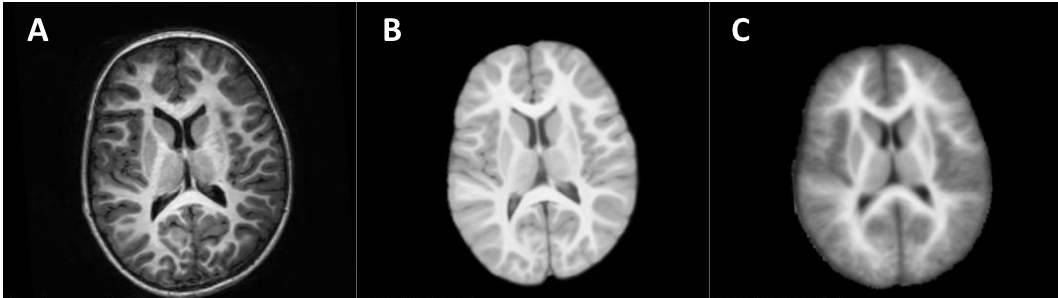


**Fig. S.4.** An example of (A) a pediatric MRI from DYSCO dataset with (B) the NIHPD 4-8 brain template and (C) the DYSCO 5-8 brain template warped to this image.

## **Comparison on GM and WM segmentation**

Our assumption was that a more similar brain template to the subject brain image will lead to a better segmentation. In order to test this assumption, we compared the segmentation accuracy obtained with the two pediatric atlases, while looking at their similarity to the image. The segmentation accuracy is measured based on the Dice overlap between the automated segmentation and the manual segmentation. As presented in **Table *S.*1**, the accuracy was not improved when the DYSCO 5-6 atlas although the similarity with the subject brain images was higher compared with the NIHPD 4-8 atlas. The segmentations obtained with the two age-specific atlases showed rather similar overlap with the manual segmentation.

**Table S.1.** Segmentation accuracy for gray matter (GM) and white matter (GM) relative to the normalized mutual information, assessed on one scan of good quality and one scan of low quality from DYSCO dataset.

| Automatic method | Dice overlap coefficient (with manual segmentation) | | | | | |
| --- | --- | --- | --- | --- | --- | --- |
|  | Scan of good quality | | | Scan of low quality | | |
|  | NMI | GM | WM | NMI | GM | WM |
| **childmetrix - NIHPD 4-8** | 0.638 | 82.05% | 82.65% | 0.647 | 83.74% | 83.66% |
| **childmetrix - DYSCO 5-6** | 0.661 | 81.81% | 82.27% | 0.673 | 83.03% | 83.88% |

## **Discussion**

When comparing the two age-specific atlases, results obtained with the study-specific atlas were not significantly improved compared to those obtained with the independent population-based atlas. This might be explained by the fact that only the brain template was built from data of the DYSCO project and not the tissue probability maps that were the same as used in the independent population-based atlas. Further investigations should be conducted in order to determine if tissue probability maps built from the studied sample might conduct to better delineation of GM and WM. In this study, building those maps was not feasible since it requires manual segmentations of GM and WM. Also, the strategy to build the study-specific atlas might not be as efficient as the one used to build the independent population-based atlas, which was sharper than the study-specific atlas. Nevertheless, as the results were similar between both age-specific atlases, we conclude that the age-specificity of the atlas is the most important feature to improve the accuracy and the reproducibility, especially for GM segmentation, rather than the study-specificity.
